# Clinical Aspects and Detection of Emerging Rickettsial Pathogens: A “One Health” Approach Study in Serbia, 2020

**DOI:** 10.3389/fmicb.2021.797399

**Published:** 2022-01-26

**Authors:** Pavle Banović, Adrian A. Díaz-Sánchez, Verica Simin, Angélique Foucault-Simonin, Clemence Galon, Alejandra Wu-Chuang, Dragana Mijatović, Dasiel Obregón, Sara Moutailler, Alejandro Cabezas-Cruz

**Affiliations:** ^1^Ambulance for Lyme Borreliosis and Other Tick-Borne Diseases, Department of Prevention of Rabies and Other Infectious Diseases, Pasteur Institute Novi Sad, Novi Sad, Serbia; ^2^Department of Microbiology With Parasitology and Immunology, Faculty of Medicine in Novi Sad, University of Novi Sad, Novi Sad, Serbia; ^3^Department of Biology, University of Saskatchewan, Saskatoon, SK, Canada; ^4^Department for Microbiological & Other Diagnostics, Pasteur Institute Novi Sad, Novi Sad, Serbia; ^5^ANSES, INRAE, Ecole Nationale Vétérinaire d’Alfort, UMR BIPAR, Laboratoire de Santé Animale, Maisons-Alfort, France; ^6^School of Environmental Sciences, University of Guelph, Guelph, ON, Canada

**Keywords:** ticks, tick-borne pathogens, coinfections, rickettsial pathogens, One Health

## Abstract

Ticks carry numerous pathogens that, if transmitted, can cause disease in susceptible humans and animals. The present study describes our approach on how to investigate clinical presentations following tick bites in humans. To this aim, the occurrence of major tick-borne pathogens (TBPs) in human blood samples (*n* = 85) and the ticks collected (*n* = 93) from the same individuals were tested using an unbiased high-throughput pathogen detection microfluidic system. The clinical symptoms were characterized in enrolled patients. In patients with suspected TBP infection, serological assays were conducted to test for the presence of antibodies against specific TBPs. A field study based on One Health tenets was further designed to identify components of a potential chain of infection resulting in *Rickettsia felis* infection in one of the patients. Ticks species infesting humans were identified as *Ixodes ricinus*, *Rhipicephalus sanguineus* sensu lato (s.l.), *Dermacentor reticulatus*, and *Haemaphysalis punctata*. Five patients developed local skin lesions at the site of the tick bite including erythema migrans, local non-specific reactions, and cutaneous hypersensitivity reaction. Although *Borrelia burgdorferi* s.l., *Babesia microti*, *Anaplasma phagocytophilum*, and *Candidatus* Cryptoplasma sp. DNAs were detected in tick samples, different *Rickettsia* species were the most common TBPs identified in the ticks. The presence of TBPs such as *Rickettsia helvetica*, *Rickettsia monacensis*, *Borrelia lusitaniae*, *Borrelia burgdorferi*, *Borrelia afzelii*, *A. phagocytophilum*, and *B. microti* in ticks was further confirmed by DNA sequencing. Two of the patients with local skin lesions had IgG reactive against spotted fever group rickettsiae, while IgM specific to *B. afzelii*, *Borrelia garinii*, and *Borrelia spielmanii* were detected in the patient with erythema migrans. Although *R. felis* infection was detected in one human blood sample, none of the components of the potential chain of infection considered in this study tested positive to this pathogen either using direct pathogen detection in domestic dogs or xenodiagnosis in ticks collected from domestic cats. The combination of high-throughput screening of TBPs and One Health approaches might help characterize chains of infection leading to human infection by TBPs, as well as prevalence of emerging rickettsial pathogens in the Balkan region.

## Introduction

Ticks (Ixodidae) are obligate blood-feeding ectoparasites of many animal hosts, including mammals, birds, and reptiles. Ticks are currently ranked second to mosquitoes as the most common arthropod vectors of disease-causing agents to humans and the first vector of vector-borne diseases (TBDs) for domestic animals worldwide, including bacterial, parasitic, and viral pathogens (de la Fuente et al., 2017). In recent decades, the global threat of emerging and/or re-emerging TBDs, along with a growing number of potential tick-borne pathogens (TBPs), has become a major problem for human and animal health (Madison-Antenucci et al., 2020). The expanding geographical distribution of several tick species caused by climate change, and anthropogenic influence on ecological systems and animal movements poses a risk of these TBDs expanding to new regions (Rosa et al., 2018). In central, western, and northern Europe, *Ixodes ricinus*, also known as the castor bean tick, is the most widespread tick species (de la Fuente et al., 2017; Rosa et al., 2018). This species has been frequently reported to infest humans and is (Milutinovic et al., 2008b) the main vector for a large variety of TBPs transmitted to both humans and animals. Other tick species, such as the ornate cow tick *Dermacentor reticulatus*, the red sheep tick *Haemaphysalis punctata*, and the brown dog tick *Rhipicephalus sanguineus* s.l., are also widely distributed across Europe where reports of tick bites by these species in human is increasing (Rubel et al., 2021).

Serbia, a country at the crossroads of central and southeastern Europe, is endemic for a great number of TBPs that comprise the most common vector-borne diseases in the Balkan Peninsula. *Ixodes ricinus* is the most common and widely distributed tick species in Serbia, where it is responsible for the transmission of Lyme borreliosis (Jovanovic et al., 2015; Berić et al., 2017; Banovic et al., 2021b), tick-borne encephalitis (Potkonjak et al., 2017; Banovic et al., 2021c), and rickettsiosis (Samardzic et al., 2008; Banovic et al., 2021a). The role of *I. ricinus* as a vector of human granulocytic anaplasmosis, human babesiosis, and tularemia has not yet been reported in Serbia. Lyme borreliosis, caused by spirochetes of the *Borrelia burgdorferi* sensu lato (s.l.) complex, is the most prevalent tick-borne zoonosis in Europe (Raileanu et al., 2017). To date, at least ten genospecies of *B. burgdorferi* s.l. complex have been described in *I. ricinus*, including *B. burgdorferi* sensu stricto (s.s.), *B. afzelii*, *B. garinii*, *B. bavariensis*, *B. spielmanii*, *B. valaisiana*, *B. finlandensis*, *B. bissettii*, *B. carolinensis*, and *B. lusitaniae* (Raileanu et al., 2017).

Data on the incidence and prevalence of human TBPs associated with *I. ricinus* are scarce in Serbia, despite being crucial to understanding the risk of human infection (Milutinovic et al., 2008a; Radulovic et al., 2011; Banovic et al., 2021b), partly because Lyme borreliosis is not a notifiable disease in Serbia. Several pathogenic and potentially pathogenic microorganisms were previously detected in *I. ricinus* removed from tick-infested individuals in Serbia (Banovic et al., 2021a,b,c). In addition, *Borrelia* spp. and *R. felis* DNA was detected in the blood of two patients who developed clinically manifested infection after tick removal (Banovic et al., 2021b). In order to gain further insights into possible relationships between emerging TBPs, clinical presentation and course following tick bites, the present study was aimed to investigate (i) the clinical symptoms observed after tick bites, and pathogen transmission in a human study group, (ii) the occurrence of major TBPs in human blood samples and the ticks collected from the same group, through a microfluidic-based high-throughput detection method, and (iii) the identification of potential chains of infection associated to TBP infection.

## Materials and Methods

### Ethics Statement

This study was approved by the ethical committee of Pasteur Institute Novi Sad (Ethical approval No. 03/2019) and conducted according to the Declaration of Helsinki and The Patient Rights Law of the Republic of Serbia. Written informed consent for publication of this clinical case report was obtained from all the patients. Household animals and their blood samples were handled in accordance with EU Directive 2010/63/EU on animal experimentation.

### Patient Recruitment and Clinical Follow Up

In 2020, a total of 650 patients attended the medical consultation at the ‘Ambulance for Lyme borreliosis and other TBDs’ of the Pasteur Institute Novi Sad to report tick bites and tick-associated infections. Only patients that got at least one tick removed within 48 h from the moment of the visit to the institute (*n* = 195) were considered for inclusion in the study. Approval for inclusion in the study was obtained from 85 patients, with a response rate of 43.6% (85/195), accounting for 13.1% (85/650) of all patients seeking consultation. Patients were requested to visit the Pasteur Institute Novi Sad 2 weeks, 4 weeks, and 3 months after the first visit to undergo a clinical examination and follow up by a medical doctor in this medical institution. Patients were also requested to report to Pasteur Institute Novi Sad if they noticed any clinical symptoms and/or sign between the requested medical appointments. Six months after the first visit, the enrolled patients were contacted via telephone to check for the existence of any signs associated to late stages of tick-borne diseases (TBDs) (e.g., rickettsiosis or Lyme borreliosis). The clinical examination included observation of the skin in general and the skin at the tick bite site in particular. Elevation of body temperature, headache, muscle pain or itching sensation at the place of previous tick infestation, as well as development of cutaneous hypersensitivity reaction were also considered in the clinical examination. If the patients developed any of the abovementioned signs or symptoms, a more specific clinical diagnostic procedure was initiated.

### Clinical Diagnosis of Tick-Borne Diseases

Diagnosis of tick-borne bacterial infection was conducted in accordance with the Guidelines of the European Society of Clinical Microbiology and Infectious Diseases guidelines (ESCMID) (Brouqui et al., 2004). Patients who developed expanding dermal redness with or without central clearing (erythema migrans) at the place of the tick bite, following incubation period of at least 72 h after the removal of the tick were clinically diagnosed with early stage Lyme borreliosis. Besides specific Lyme borreliosis manifestation (i.e., erythema migrans), other clinical signs of bacterial tick-borne infection such as development of eschare or itching redness at the site of previous tick infestation were also considered. Skin lesions that developed at the place of the tick bite after a minimum of 72 h were considered as non-specific conditions if their presentation was not matching with given case definitions of early stage Lyme borreliosis, anaplasmosis, tick-borne rickettsioses, or tularemia as described in Guidelines of ESCMID (i.e., cutaneous hypersensitive reaction and non-expanding persistent redness at the place of previous tick bite with or without itching sensation) (Brouqui et al., 2004). Patients who did not develop clinical signs related to TBD in the 6-month follow up period were considered as healthy and were discharged from the study. The molecular and serological analyses described under the Sections “Indirect Immunofluorescence and Immunoblot-Based Serological Analyses” and “Microfluidic Real-Time PCR” below were conducted solely for the retrospective confirmation of the clinical diagnosis.

### Antibiotic and Symptomatic Treatment in Patients With Clinical Signs of Tick-Borne Diseases

Patients who presented any of the abovementioned signs (i.e., erythema migrans, eschare, and non-expanding persistent redness) or symptoms (i.e., itching sensation at the skin affected by lesion) related with bacterial TBD were prescribed an antibiotic therapy including doxycycline (100 mg, Dovicin^®^, Galenika AD, Serbia) twice per day for 10 days or amoxicillin (50 mg/kg, Hemofarm A.D., Serbia) divided in three doses per day for 14 days if patient was child. The case of cutaneous hypersensitive reaction was treated with oral administration of antihistamines. The molecular and serological analyses described below were not considered in any shape or form to inform the decision of therapeutic intervention.

### Tick Collection and Morphological and Molecular Characterization

Majority of ticks were removed in local Community Health Center ambulances, while the rest were removed either in Pasteur Institute Novi Sad or by patient themselves. In the medical facilities, ticks were removed via tweezers by grasping the ticks as close to the skin as possible and pulling in upward direction. Patients most often used tweezers, but in some cases they removed ticks using only their fingers, or they picked up the tick after its spontaneous detachment. After tick removal, patients were instructed to keep it in plastic container and bring it to Pasteur Institute Novi Sad as soon as possible. Ticks attached to recruited patients were collected (*n* = 93) and taxonomically identified based on morphological features and according to standard taxonomic keys described previously (Estrada-Peña et al., 2004). Ticks were further characterized according to sex and life stage. Molecular identification of *I. ricinus*, the most common tick in Serbia, and *Dermacentor reticulatus*, were performed by microfluidic PCR including primers/probe ITS2 specific to each tick species as previously described (Michelet et al., 2014). Attachment time of collected ticks was assessed by anamnesis (i.e., information provided by the patient). When the patient was unsure about the time between the tick bite and its detection, the scutal and coxal indices were calculated and used to assess the tick feeding period as previously described (Gray et al., 2005). Attachment times of each tick were recorded. Collected ticks were placed in 70% ethanol and conserved at −80°C until DNA extraction.

### Blood Sample Collection and Sera Extraction

After written informed consent was acquired from each patient or patient’s caretakers (in the case of underage individuals), 2 ml of blood was collected in BD Vacutainer^®^ spray-coated K2EDTA tubes (BD, Oakville, CA, United States) for adults or using the BD Vacutainer^®^ Safety-Lok™ system (BD, Oakville, CA, United States) for young patients. Blood samples were stored at −80°C until DNA extraction. Blood and tick samples were labeled to allow for a pairwise analysis of pathogen detection in ticks and humans. For serological analyses (see below), one blood sample (3 ml) was collected in patients who developed lesions suggestive of rickettsial disease or Lyme borreliosis at least 3 weeks after tick removal, using BD Vacutainer^®^ SST™ Tubes (BD, Franklin Lakes, NJ, United States). Blood samples were allowed to clot at room temperature and, after centrifugation at 2000 × *g* for 10 min, the serum was extracted and stored at −80°C until further use.

### DNA Extraction

Total DNA was extracted from blood using the Nucleospin Tissue kit (Macherey Nagel, Düren, Germany) according to the manufacturer’s instructions. Ticks were homogenized using a Precellys 24 lyser/homogenizer (Bertin Technologies, Montigny-le-Bretonneux, France) at 5,500 rpm for 20 s using 2.8 mm stainless steel beads in 180 μL of lysis buffer and 25 μL of Proteinase K from the Nucleospin Tissue kit (Macherey Nagel, Düren, Germany). Homogenates were incubated for 3 h at 56°C and DNA was extracted according to the manufacturer’s instructions. Purified DNA was eluted into 50 μL elution buffer and stored at −80°C until further processing.

### DNA Pre-amplification for Microfluidic Real-Time PCR

To improve detection of pathogen DNA, total DNA was pre-amplified using the PreAmp Master Mix (Fluidigm, CA, United States) according to the manufacturer’s instructions. Primers targeting all pathogens (see next section) were pooled by combining an equal volume of each primer for a final concentration of 200 nM. The reaction was performed in a final volume of 5 μL containing 1 μL Perfecta Preamp 5X, 1.25 μL pooled primer mix, 1.5 μL distilled water and 1.25 μL DNA. The thermocycling program consisted of one cycle at 95°C for 2 min, 14 cycles at 95°C for 15 s and 4 min at 60°C. At the end of the cycling program, the reactions were diluted 1:10 in Milli-Q ultrapure water. All the pre-amplified DNA samples were stored at −20°C until needed.

### Microfluidic Real-Time PCR

To detect major TBPs [27 bacterial species (*B. burgdorferi* s.s., *B. garinii, B. afzelii, B. valaisiana, B. lusitaniae, B. spielmanii, B. bissettii, B. miyamotoi, Anaplasma marginale, Anaplasma platys, Anaplasma phagocytophilum, Anaplasma ovis, Anaplasma centrale, Anaplasma bovis, Ehrlichia canis, Neoehrlichia mikurensis, R. conorii, Rickettsia slovaca, Rickettsia massiliae, Rickettsia helvetica, Rickettsia aeschlimannii, R. felis, Bartonella henselae, Francisella tularensis, Francisella-like endosymbionts, Coxiella-like endosymbionts*, and *Coxiella burnetii*), 7 parasite species (*Babesia microti, Babesia canis, Babesia ovis, Babesia bovis, Babesia caballi, Babesia venatorum*, and *Babesia divergens*), 5 bacterial genera (*Borrelia*, *Anaplasma*, *Ehrlichia*, *Rickettsia*, and *Mycoplasma*), 3 parasite taxa (Apicomplexa, *Theileria*, and *Hepatozoon*)], the BioMark™ real-time PCR system (Fluidigm, CA, United States) was used for high-throughput microfluidic real-time PCR amplification using 48.48 Dynamic Array™ IFC chips (Fluidigm, CA, United States). These chips dispense 48 PCR mixes and 48 samples into individual wells, after which on-chip microfluidics assemble real-time PCR reactions in individual chambers before thermal cycling, resulting in 2,304 individual reactions. Briefly, amplifications were performed using 6-carboxyfluorescein (FAM)- and black hole quencher (BHQ1)-labeled TaqMan probes with TaqMan Gene expression master mix according to the manufacturer’s instructions (Applied Biosystems, Courtaboeuf, France). PCR cycling included 2 min at 50°C, 10 min at 95°C, followed by 40 cycles of two-step amplification of 15 s at 95°C, and 1 min at 60°C. One negative water control was included per chip. To determine whether factors present in the sample could inhibit the PCR, *Escherichia coli* strain EDL933 DNA was added to each sample as an internal inhibition control, and primers and a probe specifically for the *E. coli* intimin gene (*eae*) were used. For more details regarding the development of this new high-throughput tool based on real-time microfluidic PCRs (test of sensitivity, specificity, and controls used), please see Michelet et al. (2014) and Grech-Angelini et al. (2020).

### Validation of Microfluidic Real-Time PCR System Results by PCR and DNA Sequencing

In order to validate the microfluidic real-time PCR results, some positive samples to infectious agents were selected to undergo additional conventional and nested PCR assays using primers different from those of the BioMark™ system (Table 1; Regnery et al., 1991; Choi et al., 2005; Rar et al., 2005; Loh et al., 2016; Masatani et al., 2017). Amplicon sequencing was commissioned to Eurofins MWG Operon (Ebersberg, Germany) and sequences were assembled using the BioEdit software (Ibis Biosciences, Carlsbad, CA, United States). The final nucleotide sequences were analyzed to identify the sequenced microorganisms using the GenBank database through the Basic Local Alignment Sequence Tool (BLAST) search engine^1^ of the National Center for Biotechnology Information (NCBI; Bethesda, MD, United States). Nucleotide sequence data reported in the present study are available in the GenBank, EMBL and DDBJ databases under the accession numbers: MW901468-MW901476, MW901478-MW901482, MW900162, MW900163, MW900166, and MW900167.

**TABLE 1:** Set of primers used to validate microfluidic real-time PCR results.

Pathogen	Target gene	Primer sequences (5′–3′)	Name of primers	Amplicon size	References
*Anaplasma* spp.	16S rRNA	Outer primers			
		GAACGAACGCTGGCGGCAAGC	EHR1	686 bp	Rar et al., 2005
		AGTA(T/C)CG(A/G)ACCAGATAGCCGC	EHR2		
		Inner primers			
		TGCATAGGAATCTACCTAGTAG	EHR3	592 bp	
		AGTA(T/C)CG(A/G)ACCAGATAGCCGC	EHR2		
Apicomplexa	18S rRNA	Outer primers			
		GTGAAACTGCGAATGGCTCATTAC	BTH18S1stF	1500 bp	Masatani et al., 2017
		AAGTGATAAGGTTCACAAAACTTCCC	BTH18S1stR		
		Inner primers			
		GGCTCATTACAACAGTTATAGTTTATTTG	BTH18S2ndF	785	
		CGGTCCGAATAATTCACCGGAT	BTH18S2ndR		
*Borrelia* spp.	Flagellin B (*flab*)	Outer primers			
		GCAGTTCARTCAGGTAACGG	FlaB280F	645 bp	Loh et al., 2016
		GCAATCATAGCCATTGCAGATTGT	FlaRL		
		Inner primers			
		GCATCAACTGTRGTTGTAACATTAACAGG	flaB_737F	407 bb	
		ACATATTCAGATGCAGACAGAGGT	FlaLL		
*Rickettsia* spp.	Outer membrane protein B (*ompB*)	Outer primers			
		GTCAGCGTTACTTCTTCGATGC	Rc.rompB.4362p	475 bp	Choi et al., 2005
		CCGTACTCCATCTTAGCATCAG	Rc.rompB.4836n		
		Inner primers			
		CCAATGGCAGGACTTAGCTACT	Rc.rompB.4496p	267 bp	
		AGGCTGGCTGATACACGGAGTAA	Rc.rompB.4762n		
*Rickettsia* spp.	Citrate synthase (*gltA*)	Primers			
		GGGGGCCTGCTCACGGCGG	Rsfg877	381 bp	Regnery et al., 1991
		ATTGCAAAAAGTACAGTGAACA	Rsfg1258		

### Indirect Immunofluorescence and Immunoblot-Based Serological Analyses

Serological analyses were performed only in sera samples of patients who developed lesions suggestive of rickettsial disease and/or Lyme borreliosis. The tests were conducted following serum inactivation at 56°C.

Reactivity of patients IgG to spotted fever group rickettsiae (SFGR) antigens was tested using a commercial indirect immunofluorescence (IFA) assay (Vircell, Spain, Ref. ‘PRICOG’), where *Rickettsia conorii* is used as the source of SFGR antigens. Since the assay was used in a qualitative manner to detect exposure (or not) to SFGR and/or SFGR-related microorganisms (e.g., *R. felis*), only one sera dilution was used without further sera titration. The cut-off dilution 1:40 was used, according to manufacturer instructions.

*Borrelia* seropositivity was evaluated via two-tier testing. First tier test consisted of anti-*Borrelia* IgM (Euroimmun AG, Germany, Cat. FI 2138-1010-2 M) and IgG (Euroimmun AG, Germany, Cat. FI 2138-1010-2 G) detection using commercial IFA assays. Visible fluorescence reactions using sera dilutions ≥1:10 for IgM and ≥1:100 for IgG were considered as positive in the IFA. Fluorescence was visualized using a microscope (Leica DM 3000, Wetzlar, Germany) with a mercury bulb light source and N2.1 filter (Leica, Wetzlar, Germany) with an excitation wavelength of 515–560 nm. Second tier test consisted in the detection of IgM and IgG specific to the proteins p100, VlsE, p58, p41 (flagellin), p39, OspA, four strain-specific OspC proteins (from *B. burgdorferi* s.s., *B. afzelii*, *B. garinii* and *B. spielmanii*) and five strain-specific p18 antigens (from *B. burgdorferi* s.s., *B. afzelii*, *B. garinii*, *B. spielmanii* and *B. bavariensis*) using a commercial immunoblot-based assay (*recom*Line *Borrelia* IgM/IgG, Mikrogen diagnostik, Germany, Cat. 4272 and 4273). First and second tier tests were performed and analyzed according to manufacturer instructions.

### Field Studies to Identify Components of the Chain of Tick-Borne Pathogen Infection in Humans

If TBPs were detected in a patient’s blood, but not in the tick collected from that patient, a field study was organized to identify, and take samples from, components of a potential chain of infection including humans, domestic animals and ticks in defined locations were the patient could have been exposed to the detected pathogen. Collected samples were analyzed using microfluidic PCR. This approach follows the ‘One Health’ principle that health issues at the human-animal-environment interface are better understood when disease monitoring considers how diseases spread among people, animals, and the environment.

### Statistical Analysis

The percentage (%) and 95% confidence interval (CI 95%) calculations were performed using GraphPad Prism v.8.0.1 (GraphPad Software Inc., La Jolla, CA, United States). Yates continuity correction as implemented in GraphPad Prism v.8.0.1 was applied to CI 95% calculations.

## Results

### Demographic Features of Tick-Infested Patients, Characterization of Collected Ticks and Assessment of Tick Attachment Time

**FIGURE 1:**
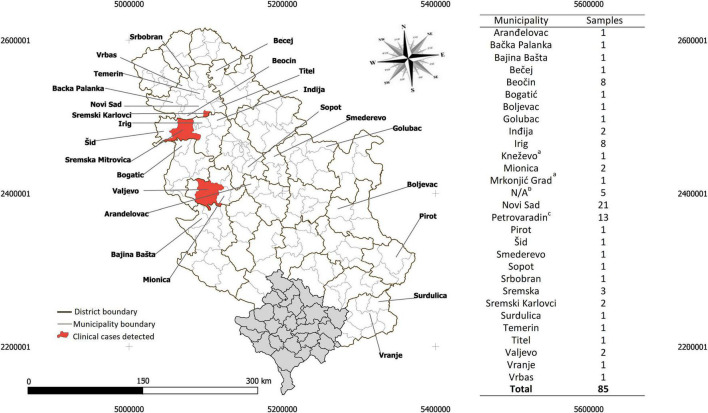
Geographical location of the patients infested by ticks in Serbia and enrolled in the study. The sample distribution is presented by municipalities, across the districts of Serbia (Supplementary Table 1). (‘a’) Two case studies from two municipalities in the neighboring country, Bosnia-Herzegovina, were labeled. (‘b’) Patients from Fruška Gora mountain with no specific municipality were labeled N/A. (‘c’) Petrovaradin is a small municipality adjacent to Novi Sad. The Serbian and Kosovo shapefile for mapping at district and municipality levels is available at the GADM database of Global Administrative Areas (v3.6, April 2020, https://gadm.org/). The map was generated using QGIS v3.12 (QGIS Development Team 2020).

A total of 85 patients with at least one tick attached were enrolled in the present study. Eighty-three (97.65%, 83/85) patients were from 27 municipalities in Serbia—mostly from the South Baèka district (59.03%, 49/83)—while two (2.35%, 2/85) patients were from the Kneževo and Mrkonjić Grad municipalities in the Banja Luka region of Bosnia and Herzegovina (Figure 1). Of the 85 patients, 80 (94.11%), 4 (4.70%), and 1 (1.18%) reported infestations with one, two, or five ticks, respectively. The ticks collected were morphologically identified to be four different species including *I. ricinus* (47 females, 34 nymphs, and 6 larvae), *R. sanguineus* s.l. (2 females and 1 male), *D. reticulatus* (2 females), and *H. punctata* (1 nymph), which were collected from 81, 3, 2, and 1 patient(s), respectively (Supplementary Table 1). The attachment time of the removed ticks were determined and resulted in the following distribution: <24 h (35.48%, 33/93), between 24 h and 48 h (26.88%, 25/93) and >72 h (31.18%, 29/93), while in 6.45% of ticks (6/93) the infestation period could not be assessed due to tick destruction during the removal process and inability of the patient to specify the moment of tick attachment. Most of the patients were between 25 and 50 years old (49.41%, 42/85), followed by patients between 50 and 75 (37.64%, 32/85) and patients less than 25 (11.76%, 10/85), while only one patient was older than 75 (1.17%, 1/85) (Supplementary Table 1).

### Skin Lesions in Tick-Infested Individuals

Overall, five out of 85 (5.88%) enrolled patients developed at least one of the clinical signs considered (Table 2). Local skin lesions at the site of the tick bite were the most frequent clinical sign observed in the five patients. Among these, patient number 597/20 (1/85; 1.18%) was diagnosed as being in the first stage of Lyme borreliosis. Accordingly, erythema migrans was identified in patient number 597/20. Three other patients (3/85; 3.53%)—identified as 225/20, 370/20 and 374/20—developed local non-specific reactions, while patient 412/20 (1/85; 1.18%) developed cutaneous hypersensitivity reaction on multiple sites of both the most recent and previous tick infestations. Detailed clinical findings in each of these patients are described below.

**TABLE 2:** Observed clinical signs.

Clinical signs observed	Clinical signs observed in patients*				
	597/20	225/20	370/20	374/20	412/20
Expanding redness at the site of previous tick infestation	X	–	–	–	–
Non-expanding redness at the site of previous tick infestation	–	X	X	–	–
Enlarged painful lymph node	–	–	–	X	–
Cutaneous hypersensitivity reaction’	–	–	–	–	X
Elevation of body temperature	–	–	–	–	–
Enlarged non-painful lymph node	–	–	–	–	–
Eschare	–	–	–	–	–

*The presence (‘X’) and absence (‘–’) of clinical signs per patient are displayed.

### Clinical Findings Associated With Lyme Borreliosis

#### Patient 597/20

**FIGURE 2:**
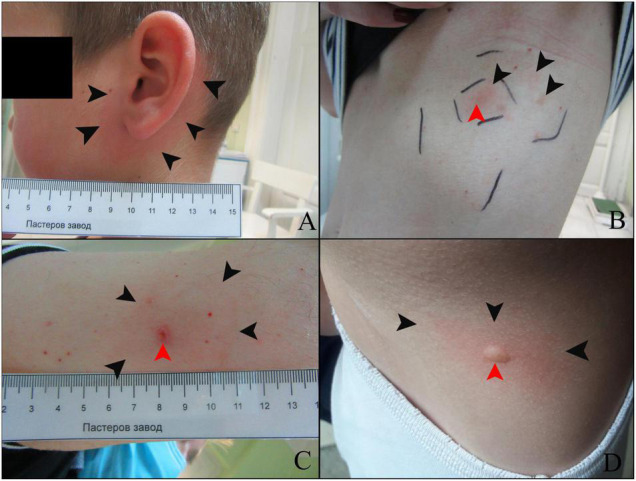
Skin lesions in patients infested with ticks. **(A)** Patient 597/20; black arrows point to the erythema migrans. **(B)** Patient 225/20; non-specific condition as consequence of tick bite encircled with outer and inner squares. Black arrows point to a non-specific focal redness, while the red arrow points to the site of the latest tick infestation. **(C)** Patient 370/20; black arrows point to a non-specific mild red lesion, while the red arrow points to the site of the latest tick infestation. **(D)** Patient 412/20; black arrows point to a reactive red area surrounding urtica (pointed to by a red arrow) at the site of a previous tick bite.

On July 31st, 2020, a 6-year-old boy was admitted to the Pasteur Institute Novi Sad with a tick attached in the left retroaurical space. The tick was identified as an *I. ricinus* nymph, with an attachment period of approximately 72 h (Supplementary Table 1). As stated by the patient’s father, 3 days earlier they had outdoor activities in the mountainous area of Divèibare, Valjevo municipality (see Figure 1). Eight days later, the boy was admitted for a medical examination due to an expanding circular redness around the site of the tick bite. At the time of examination, the diameter of the lesion was approximately 5 cm and resembled an erythema migrans (Figure 2A). No additional signs of disease were noticed. The first-tier serological examination (see methods) revealed the presence of *Borrelia*-specific IgM (dilution 1:10) and IgG (dilution 1:100) in the patient’s serum. Anti-OspC IgM specific to *B. afzelii*, *B. garinii* and *B. spielmanii* and anti-p41 IgM and IgG were detected in the second-tier test. Oral amoxicillin 50 mg/kg (Hemofarm A.D., Serbia) t.i.d. treatment for 14 days was prescribed. The erythema migrans disappeared 14 days after starting the treatment. No additional signs of disease were noticed in the 2-month follow-up period.

### Cases of Local Non-specific Skin Reactions

#### Patient 225/20

On May 14th, 2020, a 43-year-old female arrived at the Pasteur Institute Novi Sad with a tick attached to her abdomen. The tick was identified as an *I. ricinus* nymph, with an attachment period of approximately 48 h (Supplementary Table 1). The patient recalled having visited Bešenovaèko Lake on the Fruška Gora Mountain (Sremska Mitrovica municipality, see Figure 1) in that time period. On the first scheduled medical examination (June 1st) the patient had no complaints and no signs of disease were noticed. Eight days later (26 days after tick removal), the patient was admitted with an oval-shaped red lesion with prominent macula at the site of the tick bite and several discrete red spots on the periphery of the lesion (Figure 2B). The main lesion was approximately 2 cm × 4 cm in diameter. No additional signs related to this condition were noticed. The lesion was encircled with a flat tip pen and the patient was advised to report immediately if it expanded outside the drawn borders (Figure 2B). No enlargement of inguinal lymph nodes was detected during a physical examination. Five days later (31 days after tick removal), the lesion was still present, but had not expanded. A treatment of oral doxycycline 100 mg (Dovicin, Galenika AD, Serbia) twice per day for 10 days was prescribed. The lesion disappeared during the course of antibiotic treatment and the patient made a complete recovery. The patient’s serum was not reactive to SFGR antigens.

#### Patient 370/20

On June 12th, 2020, a 57-year-old man visited the Pasteur Institute Novi Sad with a tick attached to his right upper arm. The tick was identified as an adult female *I. ricinus* with an approximate attachment period of 5 days. The patient recalled having a picnic the previous weekend in Sremski Karlovci village (Sremski Karlovci municipality, see Figure 1). Two weeks after tick removal, the patient reported to the medical service of the Pasteur Institute Novi Sad that he had an itching sensation and redness at the site of the tick bite. In addition, he reported sleep deprivation and tiredness. During the physical examination, a discrete circular erythematous lesion was observed reaching 4 cm in diameter (Figure 2C). The lesion’s surface was at the same level as the non-affected skin. Scratch marks were present in the same area, while a superficial lesion in the epidermis was noticed at the site of a previous tick infestation. No enlargement of axillar lymph nodes was detected during a physical examination. The red area was then encircled with a flat tip pen and the patient was advised to report immediately if the lesion expanded beyond the drawn borders. A week later, no expansion was observed. Despite no additional signs of disease being noticed, the patient’s sensation of tiredness persisted. Since discrete redness and itching persisted, a treatment of oral doxycycline 100 mg (Dovicin, Galenika AD, Serbia) twice per day for 10 days was prescribed. The patient reported that the itching sensation intensified after the first dose of doxycycline and disappeared completely the next day. The skin lesion disappeared during the course of antibiotic treatment and the patient made a complete recovery. The patient’s serum was reactive to SFGR antigens and non-reactive to *Borrelia* antigens on first tier serology assay.

#### Patient 374/20

On June 15th, 2020, a 74-year-old female visited the medical service of the Pasteur Institute Novi Sad with an adult female *R. sanguineus* s.l. tick attached to the occipital region of her scalp. The approximate attachment time was estimated to be 72 h (Supplementary Table 1). The patient could not recall any specific activities in that time period, but mentioned contact with her dogs in nearby Žabalj village (Žabalj municipality). A week later, she was admitted with an enlarged and very painful occipital lymph node. No enlargement of the neck and mandibular lymph nodes was detected during a physical examination. A treatment of oral doxycycline 100 mg (Dovicin, Galenika AD, Serbia) twice per day for 10 days was prescribed. The pain and lymph node both decreased during the course of antibiotic treatment and the patient made a complete recovery. The patient’s serum was reactive to SFGR antigens.

### Case of Cutaneous Hypersensitivity Reaction

#### Patient 412/20

On June 16th, 2020, a 9-year-old girl was admitted with an *I. ricinus* larva attached to her left lower limb. The attachment period of the tick was estimated to be approximately 48 h. Fruška Gora Mountain (Letenka locality, Sremska Mitrovica municipality) was mentioned as the probable locality of tick aquisition. As stated by the parents, she had many tick infestations in previous years. Three weeks after tick removal, she visited the medical service of the Pasteur Institute Novi Sad with an itching sensation at the site of the last tick bite (tick included in this study) and of previous tick bites. The itching sensation was described as constant during the day but more intense during the night. During a physical examination, urticaria was noticed in several foci on the back (Figure 2D), shoulder and left lower limb at the site of the recent tick infestation. A treatment of oral loratadine 10 mg (Claritine, Schering-Plough Labo N.V., Belgium) once a day was prescribed. After 7 days, the lesions resolved and the patient made a complete recovery.

### Tick-Borne Pathogens Identified in Ticks Collected From Humans

A total of 93 tick samples collected from enrolled patients were screened for TBP DNA using a high-throughput microfluidic real-time PCR assay. Of the 93 tick samples, 63 (67.74%; 95% CI: 58.06–77.42) were positive for at least one of the tested pathogens, while 35 (37.63%; 95% CI: 27.60–47.67) harbored a single infection, and 28 (30.11%; 95% CI: 20.61–39.61) were co-infected by up to five pathogens. PCR-positive results for the presence of TBPs were detected in *I. ricinus* (70.12%, 61/87, 38 females and 23 nymphs), followed by *R. sanguineus* s.l. (33.33%, 1/3, 1 female), and *H. punctata* (100%, 1/1, 1 nymph), while no TBPs were detected in the *D. reticulatus* ticks (2 females). DNA *Rickettsia* spp. was detected in 41/93 (44.09%; 95% CI: 33.81–54.37) tick samples, followed by *Anaplasma*/*Ehrlichia* spp. in 19/93 (20.43%; 95% CI: 12.08–28.78), and *Borrelia* spp. in 18/93 (19.35%; 95% CI: 11.17–27.54), while DNA of protozoan pathogens *Babesia*/*Hepatozoon* spp. was detected in 18/93 (19.35%; 95% CI: 11.17–27.54) tick samples. Overall, 16 different pathogens with variable prevalence were identified using species-specific primers. *Rickettsia helvetica* 31/93 (33.33%; 95% CI: 23.57–43.09) was the most common TBP detected in ticks, followed by *Anaplasma phagocytophilum* 9/93 (9.68%; 95% CI: 3.56–15.80), *B. garinii* 8/93 (8.60%; 95% CI: 2.80–14.41), *B. afzelii* 7/93 (7.53%; 95% CI: 2.06–12.99), *B. spielmanii* 5/93 (5.38%; 95% CI: 0.71–10.05), *Rickettsia monacensis* 5/93 (5.38%; 95% CI: 0.71–10.05), *Rickettsia felis* 4/93 (4.30%; 95% CI: 0.10–8.50), *Borrelia miyamotoi* 4/93 (4.30%; 95% CI: 0.10–8.50), *Babesia microti* 3/93 (3.23%; 95% CI: 0.0–6.88), *B. lusitaniae* 2/93 (2.15%; 95% CI: 0.0–5.15), and *Babesia canis* 2/93 (2.15%; 95% CI: 0.0–5.15), whereas *Borrelia bissettii* 1/93 (1.08%; 95% CI: 0.0–3.21), *B. burgdorferi* s.s. 1/93 (1.08%; 95% CI: 0.0–3.21), *Babesia venatorum* 1/93 (1.08%; 95% CI: 0.0–3.21), *Neoehrlichia mikurensis* 1/93 (1.08%; 95% CI: 0.0–3.21), and *Hepatozoon* spp. 1/93 (1.08%; 95% CI: 0.0–3.21) were detected in one tick each. The occurrence of single and co-infections with TBPs in ticks is summarized in Table 3.

**TABLE 3:** Vector-borne pathogens detected in ticks from Serbia using microfluidic PCR.

Vector-borne pathogen(s)	Total	%	95% CI^a^
**Total infected ticks (≥1 pathogen)**	**63**	**67.74**	**58.06–77.42**
*Rickettsia* spp.	41	44.09	33.81–54.37
*Rickettsia helvetica*	31	33.33	23.57–43.09
*Rickettsia monacensis*	5	5.38	0.71–10.05
*Rickettsia felis*	4	4.30	0.10–8.50
*Borrelia* spp.	18	19.35	11.17–27.54
*Borrelia garinii*	8	8.60	2.80–14.41
*Borrelia afzelii*	7	7.53	2.06–12.99
*Borrelia spielmanii*	5	5.38	0.71–10.05
*Borrelia miyamotoi*	4	4.30	0.10–8.50
*Borrelia lusitaniae*	2	2.15	0.0–5.15
*Borrelia burgdorferi*	1	1.08	0.0–3.21
*Borrelia bissettii*	1	1.08	0.0–3.21
*Anaplasma* spp.	18	19.35	11.17–27.54
*Anaplasma phagocytophilum*	9	9.68	3.56–15.80
*Ehrlichia* spp.	1	1.08	0.0–3.21
*Neoehrlichia mikurensis*	1	1.08	0.0–3.21
Apicomplexa	18	19.35	11.17–27.54
*Babesia microti*	3	3.23	0.0–6.88
*Babesia canis*	2	2.15	0.0–5.15
*Babesia venatorum*	1	1.08	0.0–3.21
*Hepatozoon* spp.	1	1.08	0.0–3.21
**Single infections**	**35**	**37.63**	**27.60–47.67**
*Rickettsia* spp.	1	1.08	0.0–3.21
*Rickettsia helvetica*	16	17.20	9.39–25.02
*Rickettsia monacensis*	3	3.23	0.0–6.88
*Rickettsia felis*	1	1.08	0.0–3.21
*Borrelia* spp.	2	2.15	0.0–5.15
*Borrelia miyamotoi*	2	2.15	0.0–5.15
*Anaplasma* spp.	2	2.15	0.0–5.15
*Anaplasma phagocytophilum*	3	3.23	0.0–6.88
Apicomplexa	5	5.38	0.71–10.05
**Mixed infections**	**28**	**30.11**	**20.61–39.61**
**Mixed infections with two pathogens**	**15**	**16.13**	**8.51–23.74**
*Anaplasma* spp. + Apicomplexa	2	2.15	0.0–5.15
*R. helvetica* + *A. phagocytophilum*	2	2.15	0.0–5.15
*R. helvetica* + Apicomplexa	2	2.15	0.0–5.15
*Rickettsia* spp. + *A. phagocytophilum*	1	1.08	0.0–3.21
*R. monacensis* + *A. phagocytophilum*	1	1.08	0.0–3.21
*Anaplasma* spp. + *B. microti*	1	1.08	0.0–3.21
*Anaplasma* spp. + *B. garinii*	1	1.08	0.0–3.21
*R. helvetica* + *B. canis*	1	1.08	0.0–3.21
*B. garinii* + *B. afzelii*	1	1.08	0.0–3.21
*R. helvetica* + *A. phagocytophilum*	1	1.08	0.0–3.21
*R. helvetica* + *R. felis*	1	1.08	0.0–3.21
*B. miyamotoi* + *Rickettsia* spp.	1	1.08	0.0–3.21
**Mixed infections with three pathogens**	**7**	**7.53**	**2.06–12.99**
*B. garinii* + *B. spielmanii* + *R. felis*	1	1.08	0.0–3.21
*B. lusitaniae* + *R. monacensis* + Apicomplexa	1	1.08	0.0–3.21
*B. garinii* + *R. helvetica* + *Hepatozoon* spp.	1	1.08	0.0–3.21
*R. helvetica* + *Anaplasma* spp. + Apicomplexa	1	1.08	0.0–3.21
*R. helvetica* + *R. felis* + *B. canis*	1	1.08	0.0–3.21
*B. afzelii* + *B. garinii* + *B. spielmanii*	1	1.08	0.0–3.21
*B. afzelii* + *B. spielmanii* + *B. bissettii*	1	1.08	0.0–3.21
**Mixed infections with four pathogens**	**5**	**5.38**	**0.71–10.05**
*B. afzelii* + *B. spielmanii* + *R. helvetica* + *B. microti*	1	1.08	0.0–3.21
*B. garinii* + *B. afzelii* + *B. lusitaniae* + *Anaplasma* spp.	1	1.08	0.0–3.21
*B. burgdorferi* + *B. afzelii* + *R. helvetica* + *Anaplasma* spp.	1	1.08	0.0–3.21
*B. garinii* + *B. spielmanii* + *R. helvetica* + *B. venatorum*	1	1.08	0.0–3.21
*Borrelia* spp. + *R. helvetica* + *A. phagocytophilum* + *N. mikurensis*	1	1.08	0.0–3.21
**Mixed infections with five pathogens**	**1**	**1.08**	**0.0–3.21**
*B. garinii* + *B. afzelii* + *B. miyamotoi* + *R. helvetica* + *B. microti*	1	1.08	0.0–3.21
**Non-detected**	**30**	**32.26**	**22.58–41.96**

*^a^95% confidence interval, Yates continuity correction performed.*

The detection of *R. helvetica*, *R. monacensis*, *B. lusitaniae*, *B. burgdorferi*, *B. afzelii*, *A. phagocytophilum*, and *B. microti* was confirmed by sequencing fragments of outer-membrane protein (*ompB*) (*Rickettsia* spp.), flagellin (*flaB*) (*Borrelia* spp.), 18S rRNA (*Babesia* spp.), and 16S rRNA (*Anaplasma* spp.) genes in selected *I. ricinus* tick samples. All obtained nucleotide sequences showed >99% identity with reference strain sequences available in the GenBank database (Table 4). The sequencing analysis of the *flaB* nucleotide sequences confirmed the presence of *B. afzelii*, *B. burgdorferi*, and *B. lusitaniae*. In addition, the *ompB* nucleotide sequence confirmed the presence of *R. helvetica* and *R. monacensis* in ticks, while 16S rRNA and 18S rRNA nucleotide sequences confirmed the presence of *A. phagocytophilum* and *Candidatus* Cryptoplasma sp., and *B. microti*, respectively.

**TABLE 4:** Sequence analysis of genes in selected TBPs.

TBP species	Tick ID* (stage and sex)	GenBank	Gene	Sequence identity (%) to a reference gene in GenBank	
*R. helvetica*	34/20 (adult female)	MW901472	*ompB*	99.2	MF163037
*R. helvetica*	370/20 (adult female)	MW901475	*ompB*	100	MF163037
*R. helvetica*	372/20 (adult female)	MW901476	*ompB*	99.2	MF163037
*R. helvetica*	444/20 (adult female)	MW901478	*ompB*	99.2	MF163037
*R. helvetica*	75-2/20 (adult female)	MW901479	*gltA*	100	KY231199
*R. monacensis*	214/20 (adult female)	MW901473	*ompB*	99.2	KU961543
*R. monacensis*	293/20 (adult female)	MW901474	*ompB*	99.2	KU961543
*R. monacensis*	84/20 (adult female)	MW901480	*gltA*	100	JX003686
*R. monacensis*	248/20 (adult female)	MW901481	*gltA*	100	JX003686
*R. monacensis*	590/20 (adult female)	MW901482	*gltA*	100	MF673860
*B. lusitaniae*	76/20 (adult female)	MW901468	*flaB*	99.1	D82856
*B. burgdorferi*	335/20 (nymph)	MW901469	*flaB*	99.8	CP001205
*B. afzelii*	278/20 (adult female)	MW901470	*flaB*	99.2	CP018262
*B. afzelii*	597/20 (nymph)	MW901471	*flaB*	99.4	CP018262
*B. microti*	173/20 (adult female)	MW900162	18S rRNA	99.7	MK609547
*B. microti*	225/20 nymph	MW900163	18S rRNA	100	MK609547
*A. phagocytophilum*	359/20 nymph	MW900166	16S rRNA	100	MK239931
*Candidatus* Cryptoplasma sp.	426/20 nymph	MW900167	16S rRNA	99.2	MG924904

*The ticks ID correspond to the patients from whom they were collected as in Supplementary Table 1.

### Tick-Borne Pathogens Identified in Ticks Collected From Humans With Skin Lesions

No TBPs were detected in the ticks collected from the patients who developed cutaneous hypersensitivity reaction (i.e., patient 412/20, *I. ricinus*, 1 larva) or lymph node enlargement with *R. felis* bacteremia (i.e., patient 374/20, *R. sanguineus* s.l., 1 female). The ticks collected from patients 370/20 (*I. ricinus*, 1 female), 225/20 (*I. ricinus*, 1 nymph), and 597/20 (*I. ricinus*, 1 nymph) tested positive for *R. helvetica*, *Anaplasma* spp. + *B. microti*, and *B. afzelii* + *B. garinii* + *B. spielmanii* infections, respectively. Three pathogens—*B. microti*, *R. helvetica*, and *B. afzelii—*identified in these ticks were selected for confirmation by sequencing (Table 4).

### One Heath Approach to Search for *Rickettsia felis* in Components of a Potential Chain of Infection

Out of 85 human blood samples, only one (1.18%; 95% CI: 0.0–3.52) tested positive to TBPs. The sample collected from a patient 374/20 was PCR-positive for *R. felis orfB* and *Rickettsia* spp. *gltA* in the microfluidic system. The presence of *R. felis* was confirmed by sequencing a fragment of *R. felis ompB* nucleotide sequence (accession number MW901477), which had 99.25% identify with a *R. felis ompB* reference sequence available in GenBank (accession number MT358275). The tick removed from the skin of patient 374/20 was identified as an adult female *R. sanguineus* s.l. that was negative for the presence of any of the TBPs tested, including *R. felis*. In an attempt to identify the origin of this *R. felis* infection, a field study was organized to test for the presence of this bacterium in components of a potential chain of infection around patient 374/20.

Approximately 4 months after probable pathogen transmission, the patient was contacted and she referred two locations where the tick could have been acquired, one in Žabalj village (45.3730° N, 20.0634° E) and the other in the city of Novi Sad (45.2396° N, 19.8227° E). The patient reported having visited a weekend cottage with two hunting dogs in the Žabalj village before noticing the tick on her scalp. The patient also reported being in contact and petting nearly every day the cat of a neighbor located in the city of Novi Sad. Thus, blood samples were collected from the two dogs and, as we were not able to receive consent from the owner to take a blood sample from the cat, we used ticks collected from the cat as xenodiagnosis method for accessing the animal status as reservoir of vector-borne pathogens. During inspection of the dogs and cat, no fleas were observed on them and no tick was observed on the dogs. None of the dog blood samples tested positive for TBPs. Both ticks collected from the cat, identified as *I. ricinus* adult females (Supplementary Figure 1), were positive for *A. phagocytophilum* (confirmed by detection of *Anaplasma* spp. 16S rRNA and *A. phagocytophilum msp*2 in the microfluidic system) and *R. helvetica* (confirmed by detection of *Rickettsia* spp. *gltA* and *R. helvetica* 23S-5S internal transcribed spacer in the microfluidic system).

## Discussion

In the present study conducted in Serbia in 2020, clinical examination of atypical TBDs was combined with high-throughput molecular diagnostics, pathogen-specific serological analysis, and field studies with the aim of providing further knowledge on clinical aspects, and on the range of TBD agents in ticks attached to enrolled patients. Clinical manifestations of TBDs were registered in five cases out of a total of 85 enrolled patients. While the majority of ticks were attached to the patients for less than 24 h, all the patients who developed clinical signs of a TBD were found to have had a tick attached for more than 48 h. This finding is in accordance with available data related to the increased ability of a competent tick vector to transmit TBPs when feeding over a longer period of time (Simo et al., 2017). The results based on molecular identification showed an overall infection prevalence of 68.82% with various TBPs among the four ixodid tick species analyzed, including *I. ricinus*, *R. sanguineus* s.l., *D. reticulatus*, and *H. punctata*, which is in accordance with the average prevalence reported in a recent study from Serbia (Banovic et al., 2021b). The most prevalent TBP found in ticks was *Rickettsia* spp., with the presence and confirmation of *R. felis* and the SFGR bacteria *R. helvetica*, and *R. monacensis*. The presence of the SFGR members mentioned above have been described in *I. ricinus* ticks collected from Serbia (Banovic et al., 2021b), as well as several Eastern European countries such as Hungary (Sreter-Lancz et al., 2005), Poland (Biernat et al., 2016), Romania, and Slovakia (Mitkova et al., 2015). Tick-borne rickettsiae are considered as potentially emerging and re-emerging pathogens which can simultaneously infect humans and result in a variety of non-specific clinical symptoms (Parola et al., 2005).

In Europe, human cases of *R. helvetica* infection have been described in Austria, Denmark, France, Italy, Slovakia, Sweden, and Switzerland by serology and/or molecular-based diagnostic assays (Parola et al., 2013). Although most of the *R. helvetica* cases reported subclinical or mild non-specific symptoms, its pathogenic potential has been described in patients from Sweden that developed a clinical picture characterized by an unexplained acute febrile illness, rash, and myasthenia, and one case was even associated with fatal perimyocarditis (Nilsson et al., 1999; Nilsson, 2009). In addition, the presence of *R. monacensis* was found in *I. ricinus* ticks. This SFGR was first identified almost 20 years ago in *I. ricinus* ticks collected from a city park in Germany, and has been described as an etiologic agent of Mediterranean Spotted Fever–like illness in Spain and Italy (Jado et al., 2007; Madeddu et al., 2012). Among the cases presented, patient number 370/20 had a local non-specific reaction, which could be associated with *R. helvetica* infection. For the purpose of differential diagnosis with other pathologic entities presented as redness at the site of the tick bite (mostly erythema migrans produced by *Borrelia*), it should be considered that the erythematous lesion of this patient was not expanding in the dynamic expected for a *Borrelia* infection and that the serum of this patient was non-reactive to *Borrelia* antigens on first tier serology assay. In addition, this patient was in chronic sleep deprivation, which could be associated with immune suppression (Besedovsky et al., 2012), which in turn may increase the susceptibility to infection by facultative TBPs such as *R. helvetica*. Although the itching sensation, one of this patient’s main symptoms, can be associated with erythema migrans induced by *Borrelia* infection, itching is not considered as a specific sign of any TBD. It is noteworthy that the itching reported by this patient intensified 10 days after the beginning of the doxycycline treatment, after which patient reached complete recovery. Further investigations should address whether itching could be considered as a sign of rickettsial presence in the lesion and if so, what is the diagnostic strength of that sign for practical use. One important factor to consider in this differential diagnosis is that the tick collected from patient 370/20 was negative to all *Borrelia* spp. tested, but positive to *R. helvetica*. In addition, the tick had an attachment period of 5 days that allows for rickettsial transmission and the serum sample from patient 370/20 was found to be reactive against SFGR, confirming exposure to SFGR member.

Another case described here as a non-specific condition associated with a tick bite is the hypersensitivity reaction in a 9-year-old girl (patient 412/20) with a confirmed history of earlier tick bites. Immediate and delayed cutaneous hypersensitivity reactions in humans at the site of a recent tick bite have been described by several authors (Beaudouin et al., 1997; Burke et al., 2005; Nakahigashi et al., 2013). It is considered that such reactions are part of acquired tick resistance (ATR) that can reduce the risk of pathogen transmission from pathogen-infected ticks to hosts (Cabezas-Cruz et al., 2021). These reactions were reported to be mediated by tick-specific IgE antibodies along with dermal and perivascular infiltrates of CD8^+^ T lymphocytes and Langerhans’ cells in two adult males from Europe (Beaudouin et al., 1997). In addition, the role of basophils in the allergic reactions associated with ATR was thought to occur after exposure to tick antigens such as carbohydrate Galα1-3Gal (α-Gal) (Nakahigashi et al., 2013; Cabezas-Cruz et al., 2021). Interestingly, the case described here is the first description of cutaneous hypersensitivity reactions at various different parts of the body, including the site of the most recent tick bite. Since ATR is considered to be a systemic rather than a localized immune response (Cabezas-Cruz et al., 2021), sensitized components of the immune system may be re-targeted to the sites of previous tick infestations. This is possibly due to the presence of remnants of the tick’s mouthparts previously encapsulated in skin following a foreign body reaction (Haddad et al., 2018). In this case, the patient’s symptoms were effectively treated with an antihistamine, so the etiology of this condition should be further investigated if symptoms re-appear following new tick bites.

Patient 374/20 was positive for *R. felis* infection by PCR diagnosis. This result was confirmed by DNA sequencing analysis. The physical examination of patient 374/20 revealed an enlarged and very painful occipital lymph node without an enlargement of the neck and mandibular lymph nodes. The occurrence of rickettsiae infections in human populations from different regions of Serbia has already been described (Samardzic et al., 2008), including the report of antibodies specific to *Rickettsia typhi*, *Rickettsia akari*, and *Rickettsia conorii* antigens in human sera (Samardzic et al., 2008). Furthermore, a previous study provided molecular evidence of the presence of an *R. felis* infection in a Serbian patient who developed local skin lesions at the site of a tick bite described as atypical erythema migrans (Banovic et al., 2021b). Interestingly, none of the tested TBPs, including *R. felis*, was detected in the *R. sanguineus* s.l. tick collected from patient 374/20, which suggests that the rickettsiae infection was not acquired from the *R. sanguineus* s.l. tick. Rickettsiosis in humans caused by *R. felis* is considered to be an emerging disease since *R. felis* is considered to be closely related to SFGR members (Lai et al., 2014), and is referred to as flea-borne spotted fever (FBSF), cat flea typhus or cat flea spotted fever, due to vector preference (Perez-Osorio et al., 2008). The clinical syndrome associated with FBSF includes several symptoms that range from non-specific flu-like illness (i.e., pyrexia, arthralgia, myalgia, headache, and fatigue) to severe multi-systemic disease accompanied by a maculopapular rash, due to widespread vasculitis (Maina et al., 2012). In this case, we have confirmed seroreactivity of patient sample to SFGR antigens. Knowing that anti-*R. felis* antibodies cross react with SFGR antigens (in this case *R. conorii*) (Lai et al., 2014), we concluded that detected seroreactivity is consequence of exposure to *R. felis* and not to *R. conorii*. The biological transmission of *R. felis* via competent ixodid tick vectors remains unclear to date. The analysis of the biological transmission of FBSF is further complicated by mounting evidence of the presence of this infectious agent in more than 40 species of fleas, ticks, mites, and mosquitoes (Brown and Macaluso, 2016). Considering that antibody cross-reactivity against SFGR/SFGR-related microorganism (*R. felis*) are known to be common, a limitation of the serological assay used here is that we only utilized *R. conorii* antigen for detection of exposure to SFGR member or *R. felis* (Fournier et al., 2002; Lai et al., 2014). Another limitation is that we did not used paired sera samples to confirm seroconversion or titer growth in tested patients (Nelder et al., 2020). Thus, this limitation can be easily explained if one considers that physicians are not able to know which patient is going to develop a disease at the time when tick is removed (Wood and Artsob, 2012). In addition, all sera samples were obtained during regular check-ups before or after therapy administration.

Based on the One Health tenets applied to TBDs (Banovic et al., 2021a), we designed a field study to identify *R. felis* infection in components of a potential chain of infection in the patient’s local environment. We hypothesized that infected domestic animals could be the source of *R. felis* infection in vectors that could transmit this pathogen to patient 374/20. We found no evidence of *R. felis* infection in the tested components of a potential epidemiologic chain. Although the ticks recovered from the cat could be from different area, one should keep in mind that tick sample was used here as a xenodiagnosis method for accessing the animal status as reservoir of vector-borne pathogens. Accordingly, if the animal is active reservoir for at least one of the tested pathogens, we expected them to be present in the tick feeding on that animal regardless of tick vector competence.

*Babesia canis*, *B. microti*, and *B. venatorum* (formerly *Babesia* sp. EU1) was detected in *I. ricinus* ticks collected from humans, although the overall prevalence was low. In Serbia, *B. canis* was previously identified in dogs and *D. reticulatus* ticks (Tomanović et al., 2013; Davitkov et al., 2015), while *B. microti* and *B. venatorum* were identified in questing *I. ricinus* (Potkonjak et al., 2016). *Babesia microti* has been described as the main etiologic agent of human babesiosis in the United States, while the *B. venatorum* genospecies is one of the main species causing human infections in Europe (Krause, 2019). Interestingly, marked differences have been reported in the epidemiology of *B. microti* since in the United States this is a well-known human pathogen, while in Europe its pathogenic potential remains unclear (Moniuszko-Malinowska et al., 2016). These differences amongst *B. microti* strains from the United States and Europe might be due (de la Fuente et al., 2017) to involvement of different tick species as competent vectors in the enzootic cycle of the pathogen (Krause, 2019; Madison-Antenucci et al., 2020) to the circulation of genetically diverse species as suggested by phylogenetic studies (Nakajima et al., 2009); and/or (Rosa et al., 2018) to the underestimation of actual prevalence in humans due to the occurrence of subclinical infections or coinfection with other TBPs such as *A. phagocytophilum*, *B. burgdorferi* s.l. complex, and *Rickettsia* spp. (Parija et al., 2015). Although *B. venatorum* infection rarely occurs in humans, it has been associated with mild clinical symptoms in patients from Austria, Belgium, Italy, and Germany (Herwaldt et al., 2003; Haselbarth et al., 2007; Lempereur et al., 2015). The human babesiosis cases associated with *B. microti* or *B. venatorum* infection are being given increased importance, since the clinical presentation of disease ranges from an asymptomatic form to a life-threatening infection characterized by severe hemolysis (Poisnel et al., 2013). Therefore, *B. microti* and *B. venatorum* herein reported in *I. ricinus* ticks should be considered among infectious hazards in the Republic of Serbia.

In this study, the presence of seven genospecies of the *Borrelia* genus, namely *B. afzelii*, *B. bissettii*, *B. burgdorferi* s.s., *B. garinii*, *B. lusitaniae*, *B. spielmanii*, and *B. miyamotoi*, was confirmed in *I. ricinus* ticks removed from human skin. Although a *B. spielmanii* infection has already been indirectly confirmed in a patient with erythema migrans (Banovic et al., 2019), we herein report the occurrence of *B. bissettii* and *B. spielmanii* in *I. ricinus* ticks for the first time in Serbia. Lyme borreliosis is the most common tick-borne zoonosis in Europe, where *I. ricinus* is the main vector for transmitting the causal agents belonging to *B. burgdorferi* s.l. complex (Sykes and Makiello, 2017). When the overall *Borrelia* prevalence found in the present study is compared to results of other similar studies in Serbia, we see wide variations between localities, ranging from 10 to 40.7% (Simin et al., 2020; Banovic et al., 2021b; Stosic et al., 2021). Compared to neighboring countries of Serbia, the *Borrelia* prevalence in ticks found in our study is higher compared to that of Croatia (Tadin et al., 2016), Bulgaria (Nader et al., 2018), and Romania (Kalmar et al., 2020). In contrast, *Borrelia* spp. infection in ticks reported here is lower than infection prevalence found in ticks removed from humans in Sarajevo city (Bosnia and Herzegovina) (Lasić et al., 2020). Interestingly, the clinical evidence and the detection of three *Borrelia* spp. in the tick attached to patient 597/20, as well as the presence of specific anti-OspC antibodies against same three species (i.e., *B. afzelii* + *B. garinii* + *B. spielmanii*) confirms that this patient had a mixed *Borrelia* infection. The patient was finally considered seroreactive only in IgM class, since the presence of anti-p41 (anti-flagellin) antibodies by itself has low diagnostic value. It is known that anti-flagellin antibodies can be generated after exposure to bacteria other than *Borrelia* (e.g., *Helicobacter pylori* and *Escherichia coli*), while anti-OspC antibodies are considered to be *Borrelia*-specific (Luft et al., 1993). The fact that patient did not reached seroconversion in IgG class is probably due to timely administration of antibiotic therapy. Another explanation would be that blood sample was acquired early and before specific anti-OspC IgG have reached detectable levels. Nevertheless, it should be mentioned that a limitation of this study is that serological tests were performed only in patients displaying specific clinical signs related to possible TBDs. For that reason, we were not able to assess the frequency of positive serological status suggesting asymptomatic or subclinical TBP infection in enrolled patients. This bias could be addressed in further studies where sera samples would be acquired from enrolled patients during the first (within 48 h after tick removal) and second (4 weeks after tick removal) examinations in order to detect seroconversion against TBPs in IgM and IgG. Additionally, the response rate below 50% (85 enrolled patients out of 195 who met the “case definition” criteria), may have represented an additional source of selection bias, with possible effects on results (e.g., positivity rates for TBPs) of the study.

In addition, the presence of *A. phagocytophilum* was only detected in *I. ricinus* ticks, with an infection prevalence similar to that previously described in Serbia (Tomanović et al., 2010; Banovic et al., 2021b). The presence of *N. mikurensis* was also detected in *I. ricinus* ticks which agree with a previous report from Serbia (Potkonjak et al., 2016). In Europe, the overall prevalence rates of *N. mikurensis* in *I. ricinus* ticks range from below 1% to over 20%, and its presence has been reported in countries neighboring Serbia such as Hungary (Tomanović et al., 2010), Romania (Kalmar et al., 2016), and Slovakia (Svitalkova et al., 2016). The pathogenic potential of *N. mikurensis* has already been described in febrile patients from Germany (von Loewenich et al., 2010), Switzerland (Maurer et al., 2013), France (Boyer et al., 2021) and Sweden (Welinder-Olsson et al., 2010). Interestingly, the analysis of *Anaplasma* spp. 16S rRNA sequences led to the identification of *Candidatus* Cryptoplasma sp. in an *I. ricinus* nymph, which is its first molecular report in Serbia. This bacterium of the family Anaplasmataceae has been described as being associated with infections in green lizards (*Lacerta viridis*) and their feeding on *I. ricinus* ticks in Slovakia (Kocikova et al., 2018). Although it has been suggested that reptiles such as lizards and snakes play a role as hosts for ixodid ticks, their role in maintaining tick-borne Anaplasmataceae bacteria in the environment has never been described. This finding should therefore be interpreted with caution since further studies are necessary to determine the enzootic maintenance and circulation of *Candidatus* Cryptoplasma sp. in Serbia. In this study, the overall prevalence of *A. phagocytophilum*, *B. microti*, *B. venatorum*, genospecies of the *B. burgdorferi* s.l. complex, *N. mikurensis*, and *Candidatus* Cryptoplasma sp. was relatively low, a finding that is in accordance with previous reports from neighboring countries, which suggests widespread distribution of these TBPs in the Balkan Peninsula.

## Conclusion

Tick-infested humans are exposed to a diverse group of TBPs in Serbia. These pathogens include extracellular (i.e., *Borrelia* spp.), and intracellular bacteria (e.g., *Rickettsia* spp.) and hemoparasites (i.e., *Babesia* spp.). The frequency of detection of these microbes in ticks collected from the enrolled patients was unequal, *Rickettsia* spp. being the most common pathogen to which tick-infested humans could be exposed in Serbia. The observed health disorders associated with tick bites in humans were both infectious (cases 597/20, 370/20, and 374/20) and allergic (case 412/20), while etiology of condition recorded in patient 225/20 was possibly multifactorial. Additionally, here we presented two clinical cases caused by emerging pathogens, *R. felis* and *R. helvetica*. Although both were successfully treated, there is as great risk for them to be non-recognized by medical practitioners due to unawareness of their presence and clinical presentation. A surveillance system and joint network to facilitate rapid communication should be established to examine true prevalence and of diseases caused by emerging rickettsial pathogens in Serbia and nearby Balkan countries. The implementation of a One Health approach can be used to study the circulation of TBPs in human habitats, since a previous study conducted by Banovic et al. (2021a) showed that the active components of chains of infection are poorly characterized for TBPs affecting humans. The approach used in this study for pathogen detection led to the identification of unexpected pathogens. To the best of the authors’ knowledge, this is the first molecular evidence of the occurrence of *B. bissettii*, *B. spielmanii*, and *Candidatus* Cryptoplasma sp. in *I. ricinus* ticks feeding on humans in Serbia.

## Data Availability Statement

During the study, we generated gene sequences that were submitted to GenBank. The accession number(s) of these sequences can be found in the article (Table 4).

## Ethics Statement

The studies involving human participants were reviewed and approved by the Ethical Committee of Pasteur Institute Novi Sad (Ethical approval No. 03/2019). The patients/participants provided their written informed consent to participate in this study. Ethical review and approval was not required for the animal study because the household animals samples were collected and analyzed as part of public health intervention in order to detect possible disease spreading foci and to implement measures for pathogen control if needed. Household animals and their blood samples were handled in accordance with EU Directive 2010/63/EU on animal experimentation. Written informed consent for participation was not obtained from the owners because before any sample was collected from the animals, we consulted the owners and received their verbal approval to collect blood samples. Written informed consent was obtained from the individual(s), and minor(s)’ legal guardian/next of kin, for the publication of any potentially identifiable images or data included in this article.

## Author Contributions

PB and AC-C contributed to the conceptualization. PB, DO, and SM contributed to the methodology. DM, VS, AF-S, CG, and AD-S contributed to the validation. AD-S, PB, AF-S, CG, AW-C, and DO contributed to the formal analysis. DM, VS, PB, and AF-S contributed to the investigation. PB, DM, AC-C, and SM contributed to the resources. PB and AW-C contributed to the data curation. AD-S, PB, and AC-C contributed to the writing—original draft preparation. PB, DO, AD-S, and AC-C contributed to the writing—review and editing. DO and PB contributed to the visualization. PB, SM, and AC-C contributed to the supervision. All authors have read and agreed to the published version of the manuscript.

## Conflict of Interest

The authors declare that the research was conducted in the absence of any commercial or financial relationships that could be construed as a potential conflict of interest.

## Publisher’s Note

All claims expressed in this article are solely those of the authors and do not necessarily represent those of their affiliated organizations, or those of the publisher, the editors and the reviewers. Any product that may be evaluated in this article, or claim that may be made by its manufacturer, is not guaranteed or endorsed by the publisher.

## Footnotes

1
www.ncbi.nlm.nih.gov/blast

## Supplementary Material

Supplementary Figure 1 Dorsal (on left) and ventral (on right) views of the two engorged *Ixodes ricinus* female ticks collected from the cat.

Supplementary Table 1 Information about the geographic distribution and age of enrolled patients and ticks collected from the patients, including tick species, developmental stage, tick attachment time, and information on the integrity of the tick specimen after removal.
