# Supplementary figures and images for: Clinical Aspects and Detection of Emerging Rickettsial Pathogens: A “One Health” Approach Study in Serbia, 2020
Source: Front Microbiol. 2022 Jan 26;12:797399. doi: 10.3389/fmicb.2021.797399 (PMC8825779; doi:10.3389/fmicb.2021.797399)

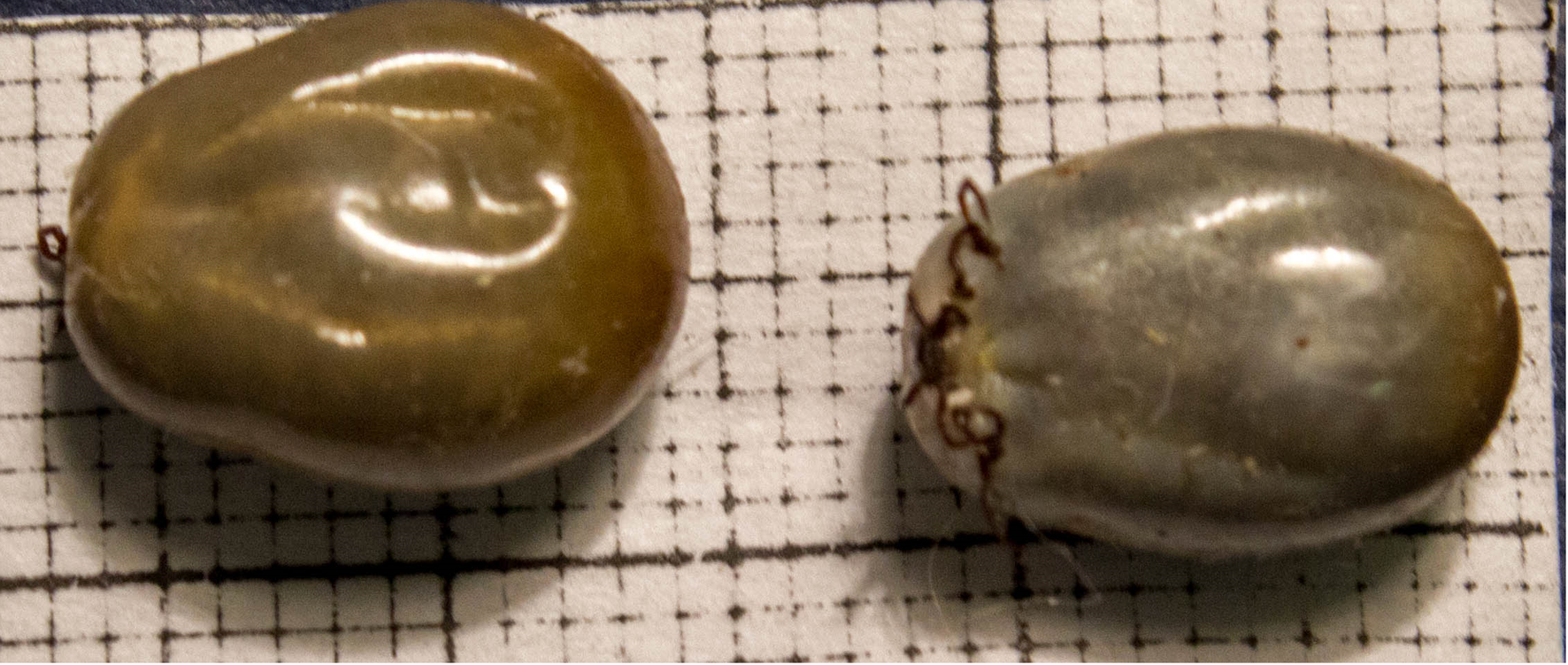

Supplement: Supplementary Figure 1 — Dorsal (on left) and ventral (on right) views of the two engorged Ixodes ricinus female ticks collected from the cat. [file Image_1.TIF]
